# Functional impairment and disability among patients with migraine: evaluation of galcanezumab in a long-term, open-label study

**DOI:** 10.1007/s11136-020-02632-0

**Published:** 2020-09-17

**Authors:** Janet H. Ford, Virginia L. Stauffer, Peter McAllister, Sreelatha Akkala, Matthew Sexson, David W. Ayer, Shufang Wang

**Affiliations:** 1 Eli Lilly and Company, 893 S. Delaware Street, Indianapolis, IN 46225 USA; 2 New England Institute for Neurology and Headache, Stamford, USA; 3 Eli Lilly Services India Pvt Ltd, Bengaluru, India

**Keywords:** Migraine, Galcanezumab, Patient reported outcomes, Migraine-specific quality of life, Migraine disability assessment, Open-label study

## Abstract

**Purpose:**

Migraine can negatively impact patient functioning and quality of life. Here, we report the effects of galcanezumab (GMB), a humanized monoclonal antibody that binds to calcitonin gene-related peptide, on patient-reported outcome (PRO) measures in migraine.

**Methods:**

CGAJ was a Phase III, randomized, open-label study (12-month open-label and 4-month post-treatment follow-up) in patients with episodic or chronic migraine. Patients aged 18–65 years with diagnosis of migraine (≥ 4 migraine headache days per month) as defined by International Classification of Headache Disorders (ICHD)-3 beta guidelines were included in the study. Patients were randomized 1:1 with subcutaneous GMB 120 mg (with a loading dose of 240 mg) or GMB 240 mg given once monthly for 12 months. Changes from baseline in PRO measures such as Migraine-Specific Quality of Life Questionnaire v2.1 (MSQ) and Migraine Disability Assessment (MIDAS) were assessed.

**Results:**

A total of 135 patients were randomized to each galcanezumab dose group. Mean (SD) baseline MSQ total scores were 53.85 (20.34) [GMB 120 mg] and 53.69 (18.79) [GMB 240 mg]. For MIDAS, mean (SD) total scores were 45.77 (42.06) [GMB 120 mg] and 53.96 (61.24) [GMB 240 mg]. Within-group mean improvement from baseline on MSQ and MIDAS total scores and all individual item/domain scores were statistically significant for both GMB dose groups, at all-time points during the treatment phase (*p* < 0.001). For MSQ domain scores, greatest improvement was observed in the Role function-restrictive (RF-R) domain (overall least squares (LS) mean change ± SE: 31.55 ± 1.20 [GMB 120 mg] and 33.40 ± 1.16 [GMB 240 mg]). For MIDAS, the overall LS mean change ± SE from baseline across the entire 12-month treatment phase in total scores were: −33.58 ± 2.11 (GMB 120 mg) and −32.67 ± 2.04 (GMB 240 mg).

**Conclusion:**

Galcanezumab was associated with statistically significant changes from baseline in the PRO measures across the entire 12-month treatment period. These results indicate improved health-related quality of life and decreased disability among patients treated with galcanezumab.

**Electronic supplementary material:**

The online version of this article (10.1007/s11136-020-02632-0) contains supplementary material, which is available to authorized users.

## Introduction

Migraine is a debilitating neurological disease with an estimated global prevalence of approximately 11.5% [1, 2]. According to the Global Burden of Disease Study, migraine is the second leading cause of years lived with disability worldwide [3]. Depending on the frequency of headache days, individuals may experience chronic migraine (≥ 15 headache days/month; of which, at least 8 have the feature of migraine headache) or episodic migraine (4–14 migraine headache days per month) [4]. The prevalence of episodic migraine is higher (11%) than chronic migraine (0.5%) [1].

The disability and functional impairment associated with migraine disrupts daily living as reported in a multi-country cross-sectional survey study of adults with migraine. This includes a strain in personal relationships, difficulty caring for children, and missed days of work or social events [5]. Notably, even a moderate attack can interfere with daily activities, often requiring bed rest [6]. The burden or impact of migraine was observed to be higher in patients with migraine compared to non-headache patients or patients with tension-type headache across domains; these included impact on work or school activities, impact on family life, interictal burden, economic burden [7]. The impact was reported to be greater in patients with increased frequency of headaches [7]. A systematic review of clinical trials and observational studies identified that there were two major determinants of decreasing the disability associated with migraine which include: (1) decreased frequency of headaches, and (2) migraine treatments [8]. Specifically, preventive medications were noted as improving work efficiency, global disability, and physical health [8]. Notably, goals of migraine prevention include reducing disability, improving patient functioning and enhancing overall health-related quality of life [9].

Commonly prescribed oral preventive medications include medicines from the antiepileptic, antihypertensives and antidepressant classes, and onabotulinumtoxinA for chronic migraine. None of these were specifically designed to treat migraine, and all are associated with high discontinuation rates, which limits the opportunity to decrease disability and improve patient functioning [10–13]. Monoclonal antibodies acting on the calcitonin gene-related peptide (CGRP) or on its receptor have emerged as new drugs specifically designed to prevent migraine, and have been noted as being plausible treatment options for reducing disability and improving functioning among patients with episodic migraine or chronic migraine by the European Headache Federation (EHF) and American Headache Society (AHS) [9, 14].

Targeting CGRP pathway has demonstrated promising results in preventive treatment of migraine [15, 16]. Galcanezumab, a humanized monoclonal antibody that binds to CGRP, is approved for the prevention of migraine. Prior phase 3 studies of galcanezumab, which analyzed patient-reported outcomes (PROs) among patients with migraine and demonstrated significant and clinically meaningful improvements in daily functioning, and migraine-related disability [17–19]. Migraine-Specific Quality of Life Questionnaire v2.1 (MSQv2.1) and Migraine Disability Assessment (MIDAS) instruments are considered as valid tools to assess patient’s health-related quality of life and disability, respectively [20, 21]. In this phase 3, open-label study, we further analyzed the migraine-specific PROs (MSQv2.1 and MIDAS) in patients treated with galcanezumab over 12 months.

## Methods

### Standard protocol approvals, registrations, and patient consents

The study protocol was reviewed and approved by institutional review boards at each study site [22]. The list of institutional review boards at each study site is provided in online resource. The study was conducted according to Good Clinical Practice and the Declaration of Helsinki guidelines. All the patients provided written informed consent, before undergoing study procedures.

### Overall study design and study objectives

Detailed study design has been described earlier [23]. The initial results of this study were presented at American Headache Society conference and the conference abstract was published [19]. Briefly, this study was a phase 3, multicenter, randomized, long-term, open-label study to assess the safety of galcanezumab 120 mg/month (with a loading dose of 240 mg) and 240 mg/month, for the treatment of episodic or chronic migraine. The study comprised of three study periods: (1) a 3- to 45-day screening period, (2) a 12-month open-label treatment phase, (3) a 4-month post-treatment period to observe the washout of the study drug. During the treatment phase, patients were randomized 1:1 to receive either 120 mg or 240 mg of subcutaneous galcanezumab once a month. Patients had to maintain a daily log of their headaches, migraine headaches and medications taken for treatment of acute episodes; this was reviewed at each monthly visit. Across the entire study, the first 3 months were clinical visits that took place at the investigator site, with both clinical (Months 6, 9, 12) and telephone visits (Months 4, 5, 7, 8, 10 and 11) at later months. During the post-treatment period, patients did not receive study medication but continued to maintain a log of their headache information and completed select patient-reported outcome measures [23].

The primary objective of the study was to evaluate the long-term safety and tolerability of galcanezumab (120 and 240 mg/month) for up to 1 year of treatment. Secondary objectives included efficacy measures such as change from baseline in number of monthly migraine headache days (MHD), headache days, frequency of medication use for acute treatment, patient’s global impression of illness/improvement, MSQv2.1 and MIDAS scores. Primary outcome and secondary efficacy outcomes that have been previously reported included improvement from baseline in MSQ RF-R domain and MIDAS total scores to month 12 [23]. This paper focuses on changes from baseline in the total score as well as each of the three domains of the MSQv2.1 for each collection point (Month 1–3, 6 and 12); and MIDAS total scores for each collection point (Month 3, 6, 9, 12) as well as changes from baseline for each item at month 12. In addition, the changes from baseline to post-treatment Months 14 and 16 for the MSQ Total and Domain scores, and Month 16 for MIDAS total scores were evaluated.

### Patient selection

Patient population consisted of males and females aged 18–65 years, with a diagnosis of migraine as defined by International Classification of Headache Disorders [24] and with a migraine onset before age 50 years. Patients who had a frequency of ≥4 migraine headache days per month (on average during the past 3 months) and a history of at least one headache-free day per month for the past 3 months were included in the study. Patients with a prior exposure to galcanezumab; use of any therapeutic antibody in the past 12 months; currently receiving medication or other treatments for prevention of migraine; history of failure to respond to three or more classes of migraine preventive treatments; history of headache other than migraine; history of traumatic head injury; recent history of acute cardiovascular events or history of stroke were excluded. Patients with concurrent tension-type headache or medication overuse headache were eligible to participate in the study.

### Outcomes measures

MSQv2.1 is a self-administered health-related quality of life status instrument that was developed to specifically address the effect of migraine on work or daily activities, relationships with family and friends, leisure time, productivity, concentration, energy, tiredness and feelings. The instrument consists of 14 items with three domain scores: Role Function-Restrictive (RF-R), Role Function-Preventive (RF-P) and Emotional Function (EF). RF-R has seven items that measure the impact of migraine on limiting individual’s social and work-related activities. RF-P has four items that measure the degree to which migraine prevents the performance of usual activities. EF has three items that measure the impact of migraine on emotions. Response options range from “none of the time" to “all of the time,” and are reverse-recoded before the domain scores are calculated. The total raw scores for each domain are transformed to a 0 to 100 scale, with higher scores indicating better functional health status [20, 25]. The instrument was designed with a 4-week recall period, and is considered reliable, valid and sensitive to change in migraine [20, 25].

MIDAS is a patient-rated scale that quantifies migraine-related disability over a 3-month period. The instrument consists of five items that reflect the number of days reported as missed/absent or with reduced productivity at work/school or home, and the number of days with missed social events. Each item has a numeric response ranging from 0 to 90 days; the days which are missed from work or home are not counted as the days with reduced productivity at work or home. The total number of days for each item are added together to produce a total score, ranging from 0 to 270, in which a higher value is indicative of more disability. Defined categorical grades of disability include Grade 1: little or no disability (0–5), Grade II: mild disability (6–10), Grade III: moderate disability (11–20), Grade IVa: severe disability (21–40) and Grade IVb: very severe disability (41 +) [21, 26, 27]

### Data analysis

The planned sample size for the study was approximately 250 patients. With the assumption of 20% dropout rate, this sample size was calculated to fulfill regulatory requirements of at least 100 patients with 1 year of exposure on each dose. The study was not powered to detect differences between two galcanezumab doses in any scales/questionnaire.

MSQ was measured at baseline and months 1, 2, 3, 6, 9 and 12 throughout the treatment period, and months 14, 16 in post-treatment period. MIDAS was measured at baseline and months 3, 6, 9 and 12 during the treatment period, and month 16 in post-treatment period. Changes from baseline in MSQv2.1 and MIDAS scores were analyzed using a restricted maximum likelihood-based mixed measures repeated model (MMRM), which included fixed categorical effects of treatment, treatment-by-visit interaction, pooled investigative site, visit, as well as the continuous fixed covariates of baseline and baseline-by-visit interaction. Analyses were conducted on an intent-to-treat basis, with patients analyzed according to assigned treatment group. P-values ≤ 0.05 were considered as statistically significant and 95% confidence intervals were provided.

## Results

Baseline demographics and disease characteristics are summarized in Table 1. Among 270 enrolled patients, 135 patients were randomized to each galcanezumab dose group. Most patients were female (82.6%) and white (78.2%) with a mean age of 42 years. Patients in the GMB 240 mg group were significantly older than those in GMB 120 mg (43.7 vs 40.2 years; *p* < 0.05). Mean baseline MIDAS total scores were numerically higher for GMB 240 mg group than the GMB 120 mg group (54.0 vs 45.8). Mean baseline MSQv2.1 total scores were similar for both dose groups (53.9 vs 53.7).

**Table 1:** Baseline demographics and disease characteristics

	GMB 120 mg *N* = 135	GMB 240 mg *N* = 135
Age, years, mean (SD)	40.2 (11.7)	43.7 (11.0)*
Sex (female), %	81.5	83.7
Race (white), %	76.3	80.0
Chronic migraine diagnosis, n (%)	26 (19.3)	31 (23.0)
MSQv2.1^†^	*N* = 133^b^	*N* = 135^b^
Total, mean (SD)	53.9 (20.3)	53.7 (18.8)
RF-R, mean (SD)	47.4 (19.2)	47.7 (18.4)
RF-P, mean (SD)	64.6 (21.7)	63.7 (21.5)
EF, mean (SD)	54.6 (29.2)	54.4 (25.3)
MIDAS	N = 133^b^	N = 135^b^
Total, mean (SD)	45.8 (42.1)	54.0 (61.2)
# days missed work or school	4.9 (11.5)	6.6 (15.1)
# days reduced productivity	9.4 (11.3)	12.5 (17.8)
# days missed household work	12.6 (15.2)	12.8 (16.2)
# days reduced productivity	11.6 (12.9)	13.0 (17.8)
# days missed family/social	7.3 (9.4)	9.1 (14.4)

^*#*^ number; ^b^Number of patients in intent-to-treat population with non-missing baseline values; *EF* emotional function; *GMB* galcanezumab; *MIDAS* migraine disability assessment; *MSQv2.1* migraine-specific quality of life questionnaire version 2.1; *RF-P* role function-preventive; *RF-R* role function-restrictive

^†^Total and each domain’s raw dimension scores were transformed to a 0–100 point scale

^*^p < 0.05 compared with 240 mg

### Health-related quality of life (MSQv2.1)

**Fig. 1:**
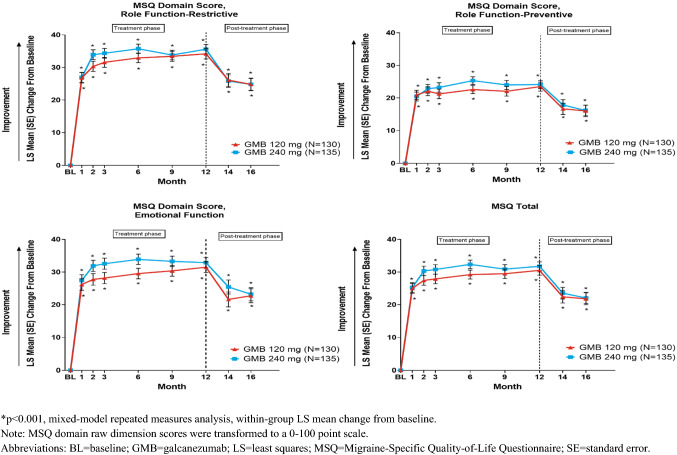
Least squares (LS) mean change from baseline ± standard error for Migraine-specific Quality of Life domains and Total scores during treatment and post-treatment phase

Within-group improvements from baseline on MSQ individual domain scores and total score were statistically significant for the GMB 120 mg and GMB 240 mg at all-time points during the treatment phase. For MSQ individual domains, significant within-group improvements (*p* < 0.001) were observed, with the greatest improvement being observed in the RF-R domain (Overall least squares (LS) mean change ± SE), 31.6 ± 1.2 (GMB 120 mg) and 33.4 ± 1.2 (GMB 240 mg) [23]. A similar trend was also observed with overall MSQ total scores during the treatment phase (28.3 ± 1.2 (GMB 120 mg) and 30.3 ± 1.1 (GMB 240 mg) [*p* < 0.001] (Table 2; Fig. 1). Overall LS mean change (SE) for MSQv2.1 individual domain scores and total score are summarized in Table 2.

**Table 2:** Changes in health-related quality of life, patient functioning and disability scores during treatment (Overall [Months 1–12])

	Overall LS mean change from baseline (SE)^a^	
	GMB 120 mg *N* = 130	GMB 240 mg *N* = 135
MSQv2.1 total		
LS mean change (SE)	28.3 (1.2)	30.3 (1.1)
Diff vs 120 mg (SE)	–	2.00 (1.6)
95% CI	–	− 1.1, 5.0
RF-R		
LS mean change (SE)	31.6 (1.2)	33.4 (1.2)
Diff vs 120 mg SE)	–	1.9 (1.6)
95% CI	–	− 1.3, 5.0
RF-P		
LS mean change (SE)	22.1 (1.1)	23.3 (1.1)
Diff vs 120 mg (SE)	–	1.3 (1.5)
95% CI	–	− 1.7, 4.2
EF		
LS mean change (SE)	28.9 (1.4)	32.0 (1.3)
Diff vs 120 mg (SE)	–	3.1 (1.8)
95% CI	–	− 0.5, 6.6
MIDAS total		
LS mean change (SE)	− 33.6 (2.1)	− 32.7 (2.0)
Diff vs 120 mg (SE)	–	0.9 (2.8)
95% CI	–	− 4.7, 6.5
MIDAS number of days missed work or school		
LS Mean Change (SE)	− 3.7 (0.5)	− 3.6 (0.4)
Diff vs 120 mg (SE)	–	0.1 (0.6)
95% CI	–	− 1.2, 1.3
MIDAS number of days with reduced productivity at work or school		
LS mean change (SE)	− 7.4 (0.6)	− 7.1 (0.6)
Diff vs 120 mg (SE)	–	0.3 (0.9)
95% CI	–	− 1.3, 2.0
MIDAS number of days missed of household work		
LS mean change (SE)	− 9.0 (0.6)	− 8.5 (0.6)
Diff vs 120 mg (SE)	–	0.5 (0.8)
95% CI	–	− 1.1, 2.0
MIDAS number of days with reduced productivity in household work		
LS mean change (SE)	− 8.4 (0.5)	− 8.0 (0.5)
Diff vs 120 mg (SE)	–	0.4 (0.6)
95% CI	–	− 0.9, 1.7
MIDAS number of days missed family or social events		
LS mean change (SE)	− 5.1 (0.5)	− 5.1 (0.4)
Diff vs 120 mg (SE)	–	− 0.1 (0.6)
95% CI	–	− 1.3, 1.2

*EF* emotional function; *GMB* galcanezumab; *MIDAS* migraine disability assessment; *MSQv2.1* migraine-specific quality of life questionnaire version 2.1; *RF-P* role function-preventive; *RF-R* role function-restrictive; *SE* standard error

^a^All p-values (within-group improvement) < 0.001

Post-treatment LS mean changes in MSQ scores from month 12 to months 14 and 16 are summarized in Table 3 and Fig. 1. During the post-treatment phase, decrease in functioning was observed. At month 14, LS mean change ± SE from month 12 in MSQ total scores were: − 7.1 ± 1.7 (GMB 120 mg) and − 8.4 ± 1.6 (GMB 240 mg) [*p* < 0.001]. Similarly, at month 16, LS mean change ± SE from month 12 in MSQ total scores were: − 8.1 ± 1.8 (GMB 120 mg) and − 9.6 ± 1.7 (GMB 240 mg) [*p* < 0.001]. Table 3 summarizes LS mean changes in individual domain scores at month 14 and 16.

**Table 3:** Changes in health-related quality of life, patient functioning and disability scores during post-treatment period from Month 12 to Month 14 and Month 16

	Baseline^a^		Month 14^b^		Month 16^b^	
	GMB 120 mg N = 106	GMB 240 mg N = 118	GMB 120 mg N = 100	GMB 240 mg N = 113	GMB 120 mg N = 99	GMB 240 mg N = 115
MSQv2.1 total						
Month 12 mean (SD) score	83.7 (18.9)	87.3 (15.5)				
LS mean change (SE)			− 7.1 (1.7)	− 8.4 (1.6)	− 8.1 (1.8)	− 9.6 (1.7)
Diff vs 120 mg (SE)			–	− 1.3 (2.2)	–	− 1.6 (2.4)
95% CI			–	− 5.7, 3.1	–	− 6.3, 3.2
RF-R						
Month 12 mean (SD) score	80.6 (20.3)	84.7 (16.4)				
LS mean change (SE)			− 7.1 (1.8)	− 9.5 (1.7)	− 8.7 (1.9)	− 10.3 (1.7)
Diff vs 120 mg (SE)			–	− 2.4 (2.4)	–	− 1.6 (2.5)
95% CI			–	− 7.1, 2.3	–	− 6.5, 3.3
RF-P						
Month 12 mean (SD) score	87.7 (16.7)	89.9 (14.8)				
LS Mean change (SE)			− 5.6 (1.6)	− 6.7 (1.5)	− 6.6 (1.7)	− 8.2 (1.6)
Diff vs 120 mg (SE)			–	− 1.1 (2.2)	–	− 1.6 (2.3)
95% CI			–	− 5.4, 3.2	–	− 6.1, 2.9
EF						
Month 12 mean (SD) score	85.5 (22.0)	90.1 (17.3)				
LS mean change (SE)			− 9.1 (2.0)	− 7.8 (1.9)	− 8.4 (2.2)	− 9.9 (2.0)
Diff vs 120 mg (SE)			–	1.4 (2.7)		− 1.5 (2.9)
95% CI			–	− 3.9, 6.6	–	− 7.2, 4.2
MIDAS total						
Month 12 mean (SD) score	15.4 (31.6)	16.1 (32.5)				
LS Mean change (SE)			–	–	7.3 (3.8)	11.8 (3.4)
Diff vs 120 mg (SE)			–	–	–	4.5 (4.8)
95% CI			–	–	–	− 5.1, 14.0

Patients did not receive any study medication during this period

*EF* emotional function; *GMB* galcanezumab; *MIDAS* migraine disability assessment; *MSQv2.1* migraine-specific quality of life questionnaire version 2.1; *RF-P* role function-preventive; *RF-R* role function-restrictive; *SD* standard deviation; *SE* standard error

^a^Baseline is the last visit of treatment phase (Month 12)

^b^All p-values (within-group improvement) for MSQ total and domain scores < 0.001

### Disability: MIDAS

**Fig. 2:**
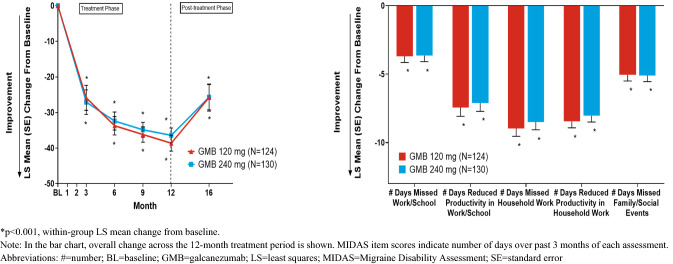
Least squares (LS) mean change from baseline ± standard error for MIDAS total score during treatment and post-treatment phase and MIDAS items during treatment phase

The within-group mean improvement from baseline on MIDAS total scores and the individual item scores were statistically significant (*p* < 0.001) for GMB 120 mg and GMB 240 mg groups, with changes ranging from very severe to a moderate or nearly moderate level of disability. Across the 12-month treatment phase, the overall LS mean change ± SE from baseline in total scores were: − 33.6 ± 2.1 (GMB 120 mg) and − 32.7 ± 2.0 (GMB 240 mg) (Table 2; Fig. 2) [23]. Individual item scores are summarized in Fig. 2.

At month 16 (5 months after treatment was stopped), increase in disability was observed; LS mean change ± SE from Month 12 in MIDAS total scores were: 7.3 ± 3.8 (GMB 120 mg) and 11.8 ± 3.4 (GMB 240 mg) [*p* < 0.001] (Table 3, Fig. 2).

### Migraine headache days

**Fig. 3:**
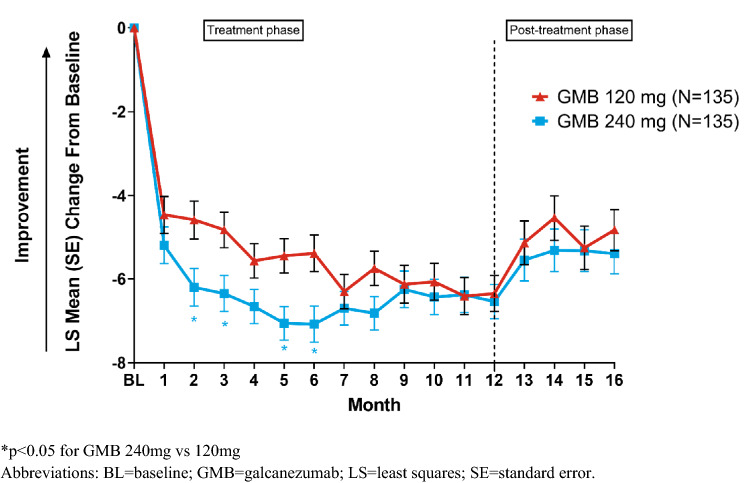
Mean changes from baseline in monthly migraine headache days during treatment and post-treatment phase

The overall reduction from baseline in the mean MHD with GMB 120 mg and 240 mg during the 12-month treatment phase has been previously reported [23]. The overall reduction from baseline in the mean MHD for GMB 120 mg and 240 mg during the 12-month treatment phase and post-treatment phase is presented in Fig. 3. During the post-treatment phase, LS mean change ± SE from baseline in MHD were: − 4.5 ± 0.5 (GMB 120 mg) and -5.3 ± 0.5 (GMB 240 mg) [*p* < 0.001] at Month 14 and − 4.8 ± 0.5 (GMB 120 mg) and − 5.4 ± 0.5 (GMB 240 mg) [*p* < 0.001] at Month 16 (Fig. 3). While the within-group reductions were statistically significant throughout the treatment and post-treatment phases (*p* < 0.001), the magnitude of the reductions decreased during the post-treatment phase relative to treatment phase.

## Discussion

### Overall findings

The study findings suggest that treatment with galcanezumab (120 mg or 240 mg), among patients with episodic and chronic migraine (≥ 4 migraine headache days per month) resulted in statistically significant and meaningful reductions in disability and improvements in health-related quality of life. These improvements were observed at month 1 and continued through month 12. At baseline, MSQv2.1 RF-R scores were less than 50, indicating considerable functional restrictions due to migraine on work and daily activities. Overall, treatment with galcanezumab up to 12 months resulted in increase of RF-R LS mean scores by 31.6–33.4 points, with significant changes from baseline scores observed as early as month 1. By month 12, the observed changes resulted in nearly 80% of the total score possible for the RF-R domain, translating into more fully functional time across work and daily activities. Similar results were observed across the other two domains which evaluated the degree to which migraine prevents the performance of daily activities and the emotional impairment associated with migraine (RF-P and EF), and for the MSQv2.1 total score that reflects improvement in overall health-related quality of life. These results indicated that galcanezumab was effective in reducing functional impairment due to migraine on work or daily activities, relationships with family and friends, leisure time, productivity, concentration, energy, tiredness and feelings beginning at month 1 and continuing through month 12.

At baseline, the study population was very severely disabled due to migraine per the MIDAS total score, and patients were absent from work/school approximately 5–7 days on average over the 3 months prior to randomization. In addition, loss of productivity was reported in nearly twice as many days (approximately 9–12 days); missed household work and productivity loss ranged from approximately 11–13 days, and family/social events were missed ~ 7 to 9 days over the prior 3 months. Over months 1–12, the level of disability (measured as LS mean change in MIDAS total score) due to migraine significantly reduced by 33.6–32.7 days, resulting in a population that had moderate to nearly moderate disability. Notably, absences and productivity losses for work/school over the last 3 months of the study were reduced by 50%. Similar or even greater reductions were observed for household work and social events.

The findings of this study are important, as the effects of galcanezumab on functional impairment and disability have only been evaluated for a duration of 6 months in patients with episodic migraine [17, 18]. The chronic migraine phase 3 study consisted of a 3-month double-blind placebo controlled phase followed by a 9-month open-label treatment phase with flexible dosing [28]. The results from our study are specific to an equal randomization to 120 mg and 240 mg doses for a full duration of 12 months in an open-label study with less frequent clinical site visits. Given that a stated treatment goal for preventive therapy is a reduction in disability and improvement in patient functioning and health-related quality of life, these galcanezumab findings that are specific to a 12-month period are clinically relevant. In addition, during the post-treatment follow-up phase, the improvement in patient’s health-related quality of life and disability decreased over time; however, the effects did remain which is expected with the half-life of the drug. These effects during post-treatment phase were consistent with previous studies [29].

Regarding similar CGRP studies, comparisons between studies are limited by differences in methodologies. The results from a phase 2 open-label extension erenumab study in patients with episodic migraine reported MSQ and MIDAS improvements from baseline for patients who had completed the 1-year open-label extension period. A decrease in approximately 15 points for the total MIDAS score and improvements of approximately 23 points for MSQ RF-R were observed [30]. Regarding chronic migraine, the efficacy of onabotulinumtoxinA was evaluated in a long-term open-label Phase IV trial, changes in how often headaches interfered with activities or caused distress was evaluated via the Headache Impact Test-6 ™ (HIT-6), demonstrating significant improvements with long-term use [31]. Overall, preventive treatments with acceptable levels of evidence per treatment guidelines are recognized as having the potential to reduce disability and improve patient functioning [9, 14].

### Strengths/limitations

As the results of this phase 3 open-label clinical study are clinically relevant, there are limitations that are worth noting. The study was not designed to evaluate the magnitude of improvement versus a comparator, or between the two dose groups of galcanezumab. The number of countries where the study was conducted in was limited; therefore, the results may not be generalizable to populations across all countries. Strengths include the use of valid and reliable patient-reported instruments that are disease specific to measure disability and health-related quality of life associated with migraine. Recall bias with the MIDAS could be seen as a confounder; however, psychometric research has demonstrated that the 90-day recall is valid and reliable, and scores are recognized as being correlated with clinical judgment as related to the need for medical care and the level of disability [26]. In addition, as this is an open-label study without placebo group, the placebo response rates that could affect the study are not considered.

These results provide important evidence for clinicians, that observed decreases in disability and improvements in health-related quality of life when treated with galcanezumab (120 mg or 240 mg) occur rapidly and are sustained over 12 months among patients with episodic and chronic migraine.

## Electronic supplementary material

Below is the link to the electronic supplementary material.

Supplementary file 1 (PDF 31 kb)

## Data Availability

Lilly provides access to all individual participant data collected during the trial, after anonymization, with the exception of pharmacokinetic or genetic data. Data are available to request 6 months after the indication studied has been approved in the US and EU and after primary publication acceptance, whichever is later. No expiration date of data requests is currently set once data are made available. Access is provided after a proposal has been approved by an independent review committee identified for this purpose and after receipt of a signed data sharing agreement. Data and documents, including the study protocol, statistical analysis plan, clinical study report, blank or annotated case report forms, will be provided in a secure data sharing environment. For details on submitting a request, see the instructions provided at www.vivli.org.
