# Supplementary material for: Functional impairment and disability among patients with migraine: evaluation of galcanezumab in a long-term, open-label study
Source: Qual Life Res. 2020 Sep 17;30(2):455–64. doi: 10.1007/s11136-020-02632-0 (PMC7886775; doi:10.1007/s11136-020-02632-0)
Supplement: Supplementary file 1 — (PDF 31 kb) [file 11136_2020_2632_MOESM1_ESM.pdf]

Functional Impairment and Disability among Patients with Migraine: Evaluation of Galcanezumab in a Long-Term, Open-Label Study

Journal: Quality of Life Research

[1]Janet H. Ford, [1]Virginia L. Stauffer, [2]Peter McAllister, [3]Sreelatha Akkala, [1]Matthew Sexson, [1]David W. Ayer, [1]Shufang Wang

[1]Eli Lilly and Company, Indianapolis, IN, USA

[2]New England Institute for Neurology and Headache, Stamford, USA

[3]Eli Lilly Services India Pvt Ltd, Bengaluru, India

Corresponding Author:

Janet H. Ford

Eli Lilly and Company

893 S. Delaware Street

Indianapolis, IN 46225, USA

Phone: 317-903-8199

Email: ford_janet@lilly.com

Supplementary material

Online resource: List of institutional review boards at each study site

Commissie Medische Ethiek Universitair Ziekenhuis [Institutes: Clinique St. Joseph, Algemeen Ziekenhuis St Jan Brugge, Universitair Ziekenhuis Brussel; Belgium], IRB Services [Institutes: DIEX Recherche Sherbrooke, Inc., Stroyan Research; Canada], Conjoint Medical Ethics Committee [Institute: University of Calgary, Canada], CPP Sud Mediterannée V [Institutes: CHU St Etienne Hôpital Nord, Hôpital de la Timone, Hôpital de Cimiez, Hôpital Lariboisiere; France], Egeszsegugyi Tudomanyos Tanacs [Institutes: Orszagos Idegtudomanyi Intezet, Petz Aladar Megyei Oktato Korhaz, SE Neurologiai Klinika, Valeomed Kft.; Hungary], Quorum Review, Inc. [Institutes: Wilmington Health Associates, Mercy Health Research, Blue Ridge Research Center, Jacksonville Center for Clinical Research, Ericksen Research and Development, Suburban Research Associates, California Medical Clinic for Headache, Albuquerque Neuroscience, Inc., ClinPoint Trials, LLC, Encompass Clinical Research, New England Institute for Clinical Research, Infinity Clinical Research, LLC, PharmQuest, Ponce School of Medicine CAIMED Center; USA]
